# Birth Weight, Childhood Body Mass Index and Risk of Coronary Heart Disease in Adults: Combined Historical Cohort Studies

**DOI:** 10.1371/journal.pone.0014126

**Published:** 2010-11-29

**Authors:** Lise Geisler Andersen, Lars Ängquist, Johan G. Eriksson, Tom Forsen, Michael Gamborg, Clive Osmond, Jennifer L. Baker, Thorkild I. A. Sørensen

**Affiliations:** 1 Institute of Preventive Medicine, Copenhagen University Hospital, Copenhagen, Denmark; 2 Department of General Practice and Primary Health Care, University of Helsinki, Helsinki, Finland; 3 Unit of General Practice, Helsinki University Central Hospital, Helsinki, Finland; 4 Folkhälsan Research Centre, Helsinki, Finland; 5 Vasa Central Hospital, Vasa, Finland; 6 Department of Health Promotion and Chronic Disease Prevention, National Institute for Health and Welfare, Helsinki, Finland; 7 Vasa Health Care Center, Vasa, Finland; 8 MRC Lifecourse Epidemiology Unit, University of Southampton, Southampton General Hospital, Southampton, United Kingdom; Indiana University, United States of America

## Abstract

**Background:**

Low birth weight and high childhood body mass index (BMI) is each associated with an increased risk of coronary heart disease (CHD) in adult life. We studied individual and combined associations of birth weight and childhood BMI with the risk of CHD in adulthood.

**Methods/Principal Findings:**

Birth weight and BMI at age seven years were available in 216,771 Danish and Finnish individuals born 1924–1976. Linkage to national registers for hospitalization and causes of death identified 8,805 CHD events during up to 33 years of follow-up (median = 24 years) after age 25 years. Analyses were conducted with Cox regression based on restricted cubic splines. Using median birth weight of 3.4 kg as reference, a non-linear relation between birth weight and CHD was found. It was not significantly different between cohorts, or between men and women, nor was the association altered by childhood BMI. For birth weights below 3.4 kg, the risk of CHD increased linearly and reached 1.28 (95% confidence limits: 1.13 to 1.44) at 2 kg. Above 3.4 kg the association weakened, and from about 4 kg there was virtually no association. BMI at age seven years was strongly positively associated with the risk of CHD and the relation was not altered by birth weight. The excess risk in individuals with a birth weight of 2.5 kg and a BMI of 17.7 kg/m^2^ at age seven years was 44% (95% CI: 30% to 59%) compared with individuals with median values of birth weight (3.4 kg) and BMI (15.3 kg/m^2^).

**Conclusions/Significance:**

Birth weight and BMI at age seven years appeared independently associated with the risk of CHD in adulthood. From a public health perspective we suggest that particular attention should be paid to children with a birth weight below the average in combination with excess relative weight in childhood.

## Introduction

The developmental origins of health and disease (DOHaD) hypothesis proposes an increase in coronary heart disease (CHD) risk in later life with decreasing birth size [1], [2]. These findings have been replicated in several populations around the world in both sexes – also within the Nordic countries [3]–[9].

In addition to non-optimal prenatal growth, postnatal growth [1], [5], [10], [11] has been associated with CHD risk. On the other hand, attained body weight (for height) later in childhood [4], [11]–[14] and young adulthood [15]–[17] has been associated with a significant increase in later CHD risk. High birth weight is associated with an increased risk of overweight in childhood [18], so it could be expected that the inverse association between birth size and CHD risk would be weakened or even reversed at higher birth weights. Some studies suggested such an effect [7], [9], [14], [17], [19], but most previous studies have not consistently identified a birth weight-CHD association compatible with this effect [2]–[6], [8], [12], [20]–[22], and it was not addressed in a recent review of the literature on birth weight and the risk of ischemic heart disease [8].

Some studies have investigated the combined effects of birth weight and later body weight on CHD risk [4], [5], [11], [12], [14], [17], [22]. Studies from Finland have found that adults who developed CHD were small at birth, thin in infancy, and thereafter rapidly put on weight reaching average body mass index (BMI; weight/height^2^; kg/m^2^) during late childhood, and the risk was determined more strongly by the rate of weight gain than the body weight attained [5], [11], [12], [21]. It remains unclear whether the shape and strength of the association between birth weight and risk of CHD depends on childhood body weight [4], [5], [11], [12], [14], [17], [22]. The present study addresses this aspect in two large populations allowing exploration of associations of combinations of birth weights and childhood BMI with risk of CHD in adults.

## Methods

### Study population

Eligible cohorts were cohorts participating in a Nordic longitudinal epidemiologic research program “Prenatal and Childhood Growth in Relation to Cardiovascular Disease” (the NordNet Study) [23]. The NordNet Study group consists of researchers from 13 study centres in Denmark, Finland, the Faroe Islands, Iceland, Norway, and Sweden who provided access to data from Nordic population-based studies of individuals constituting prospectively and/or retrospectively established cohort studies.

Among these cohorts, the research question posed here could be addressed in one large cohort of Danish schoolchildren who ever attended school in the municipality of Copenhagen [14], [24] and a cohort of Finnish men and women born either in Helsinki University Central Hospital or in the Helsinki City Maternity Hospital [6], [10], [25]. These cohorts included data on birth weight together with BMI at seven years of age and incidence of CHD in adulthood (Table 1). The Danish cohort comprised all schoolchildren born between 1936 and 1976, who underwent mandatory annual health examinations at public or private schools in Copenhagen and were resident in Denmark in 1968 from which point onwards a unique personal identification number was assigned to all Danish residents. Finnish participants were born between 1924 and 1944 at Helsinki University Central Hospital or born at the Helsinki City Maternity Hospital between 1934 and 1944, went to school in the city of Helsinki and were still resident in Finland in 1971, when all Finnish residents were assigned a unique personal identification number. The cohorts have been described in detail elsewhere [6], [10], [14], [24], [25]. The present analysis was approved by the Danish Data Protection Agency (Datatilsynet) and by the ethical committee of the National Public Health Institute, Helsinki. According to Danish and Finnish laws, informed consent is not required for register-based research of pre-existing personal data.

**Table 1:** Cohort and sex-specific characteristics of studies included in the analysis of the association between birth weight and later risk of coronary heart disease.

Country and cohort	Denmark		Finland	
Sex	M	F	M	F
**Birth years**	1936–1976	1936–1976	1924–1944	1924–1944
**Number of subjects**	102,437	97,650	8,727	7,957
**Birth weight; mean±SD**	3.43±0.55	3.31±0.53	3.47±0.49	3.34±0.46
**BMI at 7 years; mean±SD**	15.5±1.2	15.4±1.4	15.5±1.1	15.4±1.3
**Age at entry; mean±SD**	29.1±4.8	29.0±4.8	34.5±6.4	34.8±6.5
**Age at entry; range**	25, 41	25, 41	26, 47	26, 47
**Years at risk; sum**	1,934,770	1,880,211	223,699	216,149
**Years at risk; median**	23	24	25	25
**Years at risk; range**	<1, 25	<1, 25	<1, 33	<1, 33
**CHD events**	5,132	1,974	1,296	403

### Assessment of variables

Information on birth weight in the Finnish cohort was abstracted from hospital birth records, whereas in the Danish cohort the mothers reported it at the first health examination after school entry at approximately seven years of age. Birth weight (in kg) was used as a continuous variable, and the range of birth weight was restricted to 2.0 to 5.5 kg to avoid analyses of outliers due to errors in reporting, recording, or to pregnancy-related and fetal morbidities.

Body height and weight were prospectively measured at school health examinations and recorded in school health records, and were used for calculation of BMI ([weight (kg)]/[height (m)^2^]). BMI at the exact age of seven years was obtained by interpolation between successive measurements around that age or by extrapolation [4], [14].

### Assessment of outcome

Information on CHD events was obtained by linking the unique personal identification numbers of the cohort participants to various computerised, comprehensive national hospital discharge and cause-of-death registers in each country. In the Danish cohort, discharge diagnoses and causes of death were classified according to International Classification of Diseases, Eighth Revision (ICD-8) from 1977 to 1993 and according to ICD-10 from 1994 until 2001. In the Finnish cohort, causes of hospital discharges or deaths were recorded according to ICD-8 from 1971 to 1986; thereafter, ICD-9 was used until 1995 and ICD-10 until 2003. CHD was defined as ICD-8 and ICD-9 codes 410.0 to 414.9 and ICD-10 codes I20.0 to I25.9. The coverage of each of the Danish registers is very high, with virtually every event recorded [26], [27]. In the Hospital Discharge Register, myocardial infarctions are recorded with a high degree of validity [28], but in the Cause of Death Register, cardiovascular disease diagnoses are less accurate [27]. The diagnoses of acute myocardial infarction and death from CHD in the Finnish National Hospital Discharge Register and the Cause of Death Register are highly predictive, with very few false positive findings of a true major coronary event defined by strict criteria. The sensitivity of the Hospital Discharge Register, however, is only moderate. Combining the Discharge Register data with routine mortality statistics accurately reflects the occurrence of CHD in Finland [29], [30]. Thus, their use in endpoint assessment in epidemiological studies is justified. Any misclassification is likely to be independent of birth weight and lead to an underestimation of the effect.

The minimum age at study entry was set to 25 years. This age was chosen as the earliest entry point to restrict the analyses to adult CHD events. The follow-up of cohort participants ended on the date when a CHD event occurred, at the date of death, emigration, or disappearance, or at end of the follow-up period, which was 2001 for the Danish cohort, 1995 for Finnish individuals born 1924 through 1933, and 2003 for Finnish individuals born 1934 through 1944, whichever came first within each cohort.

### Statistical analyses

We examined hazard ratios (HRs) for the association between birth weight and CHD with and without adjustment for BMI at age seven years using Cox's proportional hazard regression. Age was used as the underlying time scale [31]–[33]. Potential interactions between birth weight and sex, cohorts, and BMI at age seven years respectively were assessed by the Wald test separately for sex, cohort, and BMI strata, the latter defined by tertiles or by the <10^th^, 10^th^ to 90^th^, and >90^th^ percentiles. Potential nonlinearity in the birth weight-CHD association was assessed by restricted cubic spline regression based on four knots of birth weight. These were located at the 5^th^, 35^th^, 65^th^ and 95^th^ percentiles [34], [35], which correspond to birth weights of 2.5 kg, 3.2 kg, 3.5 kg and 4.3 kg. For illustration purposes, the median birth weight of 3.4 kg was used as the reference weight; hence it corresponds to HR = 1. Potential mediating effects of BMI were investigated by comparing the shape of the unadjusted birth weight-CHD association to the BMI adjusted association. BMI was included continuously as a restricted cubic spline based on four knots as adjustment variable in the Cox- regression model. The knots were located at the 5^th^, 35^th^, 65^th^ and 95^th^ percentiles corresponding to BMI values of 13.6 kg/m^2^, 14.9 kg/m^2^, 15.8 kg/m^2^ and 17.7 kg/m^2^. Estimates of the CHD risk associated with BMI at age seven years were extracted from this model and examined with and without adjustment for birth weight. Median BMI (15.3 kg/m^2^) was used as the reference value. The correlation between birth weight and BMI at age seven years was estimated by Pearson's correlation coefficient, and the combined effect of the two was estimated by Cox's proportional hazards regression including quadratic terms for birth weight and BMI and an interaction term between birth weight and BMI.

Sensitivity analyses were conducted after further restriction of the range of birth weight to 2.5–5.0 kg and to 2.75–4.75 kg due to low numbers of CHD events, which gave essentially the same results. Estimation was conducted allowing for separate baseline hazards within each of the four cohort-sex strata, which gave similar results as adjustment for these factors. The proportional hazards assumption was assessed by visual inspection, and tested by use of Schoenfeld residuals [36]. P-values below 0.01 were considered statistically significant. All analyses were conducted using STATA version 9.2 (StataCorp LP; July 2007).

## Results

Birth weight and BMI characteristics were similar among Danish and Finnish subjects and age at study entry and years of follow up were similar across cohort and sex (Table 1). Men experienced relatively more CHD-events than women, but the distributions of birth weights and of CHD events by birth weight were fairly similar among men and women (Table 2). The tests for interaction with cohort and sex were not statistically significant (P values were between 0.09 and 0.40). Thus the presented estimated HR's are based on the pooled data for both of these factors, which are used as adjustment variables. Adjustment for year of birth did not change the estimates, which are shown unadjusted for year of birth. Also, the tests for interaction between birth weight and BMI at age seven years on the HR for CHD were not statistically significant (P values were between 0.18 and 0.40).

**Table 2:** Distribution of individuals and of CHD-events by sex and birth weight.

	Men		Women		Total	
Birth weight; kg	% (N)	% (CHD-events)	% (N)	% (CHD-events)	% (N)	% (CHD-events)
2.00–2.50	6.2 (6,887)	6.8 (440)	8.2 (8,643)	9.3 (221)	7.2 (15,530)	7.5 (661)
2.51–3.00	18.2 (20,244)	19.0 (1,223)	23.8 (25,101)	24.2 (576)	20.9 (45,345)	20.4 (1,799)
3.01–3.50	37.1 (41,296)	38.1 (2,446)	39.4 (41,657)	38.8 (923)	38.3 (82,953)	38.3 (3,369)
3.51–4.00	27.3 (30,350)	25.5 (1,637)	21.9 (23,169)	20.7 (491)	24.7 (53,519)	24.2 (2,128)
4.01–4.50	9.0 (10,005)	8.3 (536)	5.4 (5,741)	5.5 (130)	7.3 (15,746)	7.6 (666)
4.51–5.00	1.8 (1,971)	1.7 (110)	1.0 (1,029)	1.3 (31)	1.4 (3,000)	1.6 (141)
5.01–5.50	0.4 (411)	0.6 (36)	0.3 (267)	0.2 (5)	0.3 (678)	0.5 (41)
**Total**	**100 (111,164)**	**100 (6,428)**	**100 (105,607)**	**100 (2,377)**	**100 (216,771)**	**100 (8,805)**

**Figure 1:**
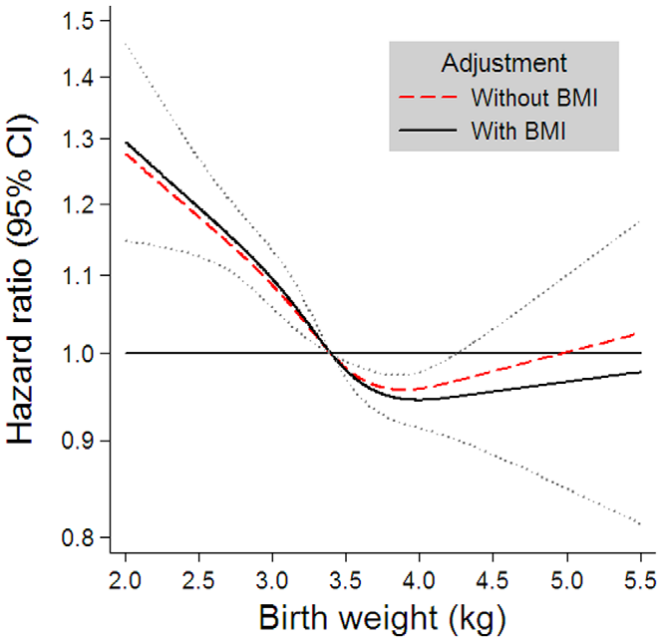
Risk of coronary heart disease in adulthood according to birth weight. The estimates are adjusted for sex and cohort (Danish or Finnish), estimated by Cox regression based on restricted cubic splines with age as the underlying time scale. The dashed line shows the estimates without adjustment for BMI at age seven years, the bold line shows the estimates with adjustment for BMI at age seven years and the dotted lines show the 95% confidence limits for the adjusted estimates.

Figure 1 shows the main result based on the pooled data. An inverse association between birth weight and the HR for CHD was observed for birth weights below 3.4 kg, whereas the association weakened above 3.4 kg, and from 4 kg through 5.5 kg there was virtually no association, although with a steady widening of the confidence intervals. The HR for CHD reached 1.28 (95% confidence interval: 1.13 to 1.44) at 2 kg. Both the test for a general birth weight-effect of any kind, and the test for a non-linear effect were highly significant after adjustment for BMI at age seven years (p<0.0005 and p = 0.009, respectively).

**Figure 2:**
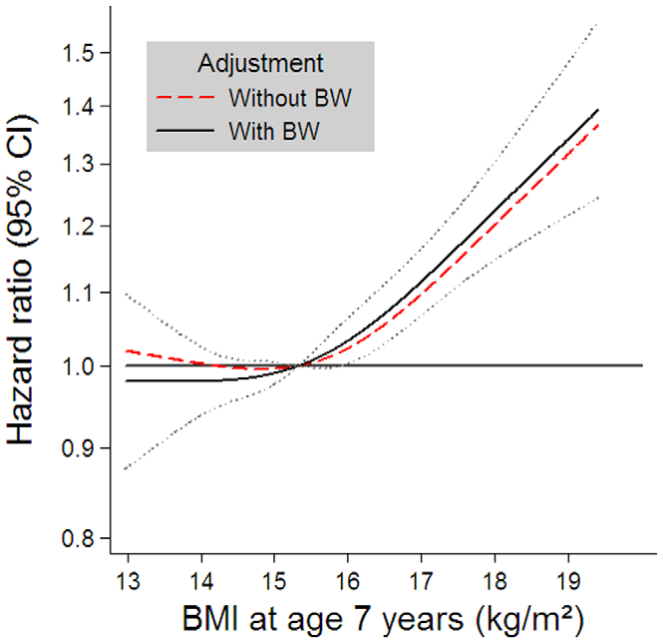
Risk of coronary heart disease in adulthood according to BMI at age seven years. The estimates are adjusted for sex and cohort (Danish or Finnish), estimated by Cox regression based on restricted cubic splines with age as the underlying time scale and truncated to depict the inner 98% of the BMI distribution. The dashed line shows the estimates without adjustment for birth weight, the bold line shows the estimates with adjustment for birth weight and the dotted lines show the 95% confidence limits for the adjusted estimates.

As expected, there was a highly significant positive association between BMI at seven years and the HR for CHD, also after adjustment for birth weight (p<0.0005). The association appeared monotonic and slightly non-linear (p = 0.03) (Figure 2). However, as seen in Figure 1, adjustment for BMI at seven years of age in the association between birth weight and the HR for CHD changed the main estimates for birth weight only minimally, and the significance level of the birth weight-CHD association remained the same after adjustment.

**Figure 3:**
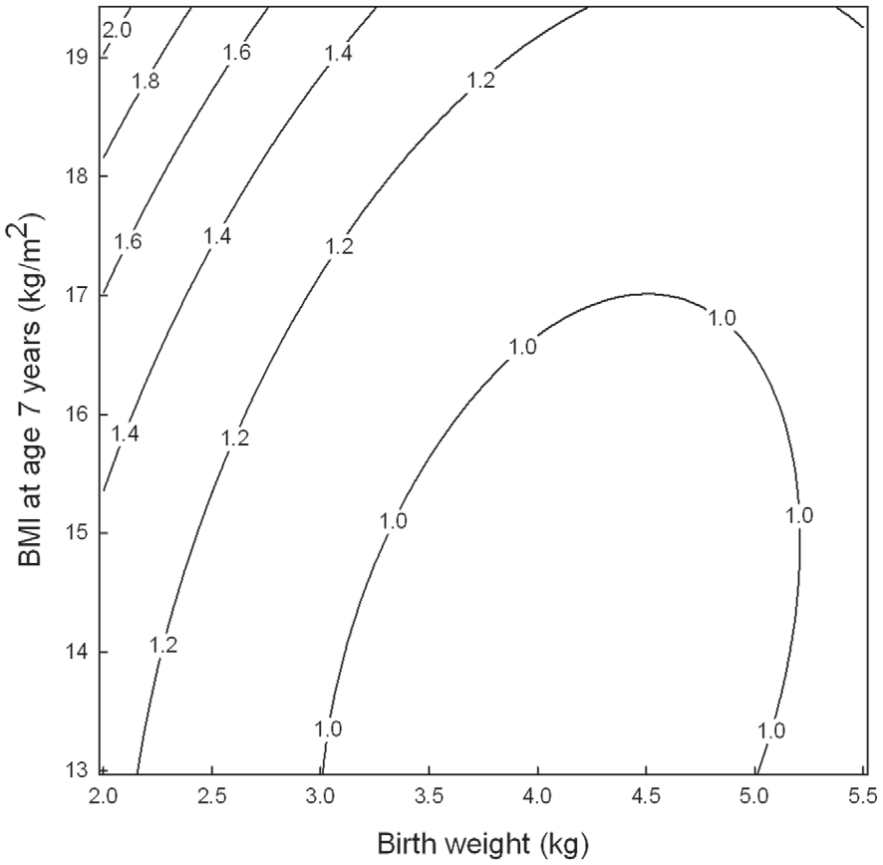
Risk of coronary heart disease according to birth weight and BMI at age seven years. The estimates are adjusted for sex and cohort (Danish or Finnish), estimated by Cox regression with age as the underlying time scale. The lines join points with the same hazard ratio for CHD and are truncated to depict the inner 98% of the BMI distribution. Arrows indicate median values, the plus indicates the risk at the joint median and the M indicates the minimum risk.

Birth weight and BMI at age seven years were weakly correlated (r = 0.18). The distribution of individuals across combinations of intervals of birth weight and BMI at age seven years is shown in table 3. The simultaneous effect of the two on the HR for CHD is illustrated in Figure 3 where the lines join points with the same hazard ratio for CHD for different combinations of birth weight and BMI at age seven years. The values are risks relative to the value of 1 for those with median birth weight (3.4 kg) and median BMI at seven years (15.3 kg/m^2^), which is marked by a +. The figure shows that the HR for CHD increased with decreasing birth weight and with increasing BMI in an independent manner. The lowest estimated HR was found for subjects with a combination of low BMI (13.7 kg/m^2^) and a birth weight of 4.1 kg (HR = 0.92 [95% CI: 0.86 to 1.00]) indicated by an M in the figure. In the upper left corner of the figure, delimited by a birth weight below 3251 g and a BMI at age seven years above 18.35 kg/m^2^, the observed HR was in the range from about 1.25 to about 2.0 (Figure 3). However, this risk pertained to only 0.8% of the population (Table 3). As an example, a child with a combination of a birth weight of 2.5 kg (the 5^th^ percentile) and a BMI of 17.7 kg/m^2^ (the 95^th^ percentile) had a risk increase of CHD of 44% (HR = 1.44 [95% CI: 1.30 to 1.59]).

**Table 3:** Distribution of individuals (% (N)) across combinations of intervals of birth weight and BMI at age seven years.

BMI at 7 years; kg/m^2^			Birth weight; g				
	2000–2750	2751–3250	3251–3750	3750–4250	4251–4750	4751–5500	Total
**>19.42**	0.1 (204)	0.2 (523)	0.4 (791)	0.2 (473)	0.1 (142)	<0.1 (30)	1.0 (2,163)
**>18.35–19.42**	0.1 (303)	0.4 (899)	0.6 (1,322)	0.3 (743)	0.1 (248)	<0.1 (61)	1.7 (3,567)
**>17.27–18.35**	0.4 (883)	1.3 (2,772)	2.0 (4,243)	1.2 (2,527)	0.4 (770)	0.1 (211)	5.3 (11,406)
**>16.19–17.27**	1.4 (2,947)	4.1 (8,983)	6.1 (13,104)	3.4 (7,320)	0.9 (2,043)	0.2 (507)	16.1 (34,904)
**>15.12–16.19**	3.5 (7,641)	9.7 (20,978)	12.5 (27,168)	5.8 (12,521)	1.4 (2,991)	0.3 (697)	33.2 (71,996)
**>14.05–15.12**	4.5 (9,808)	10.7 (23,128)	11.3 (24,546)	4.2 (9,182)	0.8 (1,708)	0.2 (398)	31.7 (68,770)
**>12.97–14.05**	2.0 (4,340)	3.8 (8,213)	3.2 (6,907)	0.9 (1,927)	0.1 (296)	0.1 (108)	10.1 (21,791)
**< = 12.97**	0.3 (567)	0.4 (869)	0.3 (551)	0.1 (166)	<0.1 (15)	<0.1 (6)	1.0 (2,174)
**Total**	12.3 (26,693)	30.6 (66,365)	36.3 (78,632)	16.1 (34,805)	3.8 (8,213)	0.9 (2,018)	100 (216,771)

The lowest and highest BMI groups represent the group below the 1^st^ and above the 99^th^ BMI percentile respectively. The inner 98% of the BMI distribution corresponding to what is shown in figure 3 is divided into six groups of equal range of BMI units.

## Discussion

We found that the risk of CHD events was inversely related to birth weight among individuals with a birth weight less than 3.4 kg, whereas for individuals with a birth weight above 3.4 kg the association weakened and levelled off from 4 kg and upwards through 5.5 kg. While the association between BMI at seven years and later risk of CHD was strong, highly significant and independent of birth weight as previously reported for the Danish cohort [14], the association with birth weight was essentially unchanged when BMI at age seven years was included in the analyses. There were no indications that the association between birth weight and the risk of CHD was modified or mediated by childhood BMI. The levelling off of the inverse association between birth weight and CHD risk could therefore not be attributed to the direct relation of high birth weight and CHD to childhood BMI. Thus, birth weight and BMI at seven years of age appear to exercise mutually independent effects on the risk of CHD.

The findings were robust. They were essentially the same for men and women and for both cohorts studied. The cohorts constituted a larger number of individuals followed up for a longer time than any of the previous studies [4], [5], [11], [12], [17], and neither the selection of individuals for the cohorts, nor the follow-up procedures leave suspicion of selection biases. Eighty eight percent of the school health cards for Danish schoolchildren could be linked to the National Civil Register. The main reasons for non-linkage were emigration, death, and changes in women's surnames before 1968 [14]. Of the Finnish subjects who were born and grew up in Helsinki, the great majority were traced and found living in Finland in 1971 (92% in the older cohort and 83% in the younger cohort) [6].

It was not the major objective of this study to address the association between BMI at seven years and risk of CHD in adulthood, which has been carefully investigated and reported on previously on the basis of the large Danish cohort [24]. It was shown that the risk of CHD later in adult life increases with BMI at these ages, more so the older the child is [14]. Among seven-year-old boys, the HR for CHD in relation to a 1-unit increase in BMI z-score was 1.09 (95% CI: 1.05 to 1.12), and among girls, 1.04 (95% CI: 1.00 to 1.09). As confirmed here, it has also been reported that these associations are not modified by birth weight in the Danish cohort [14]. However, results on the association of birth weight with later risk of CHD were not the main focus of the previous study, which did not report on potential mediating effects by BMI.

The combined effects of birth weight and weight during childhood on CHD risk have been investigated in some previous studies. One study supports the present finding that the birth weight association with CHD risk is independent of childhood BMI; an analysis of the Aberdeen children of the 1950s cohort showed that adjustment for BMI at school entry did not change the inverse association between birth weight and CHD [22]. We found that the risk of CHD fell with increasing birth weight and rose with increasing BMI. The relative importance of the two factors for later CHD risk appears to be of similar magnitude. We observed that the risk increase was about 20% for children with a birth weight at the 5^th^ percentile (2.5 kg) or with a BMI of the 95^th^ percentile (17.7 kg/m^2^) compared to children with median birth weight and median BMI, respectively (Figure 1 and Figure 2). Children with a combination of low birth weight and relatively high BMI had a risk increase of CHD of 44% (95% CI: 30% to 59%) (Figure 3). Similarly, in a Finnish cohort, it has been shown that body size at birth and weight gain from age three through 11 years had independent effects and that the combination of the two predicted large differences in the cumulative incidence of CHD (from 5.9% to 11.3%) [37]. Other studies suggest that there is an interaction between size at birth and later body weight in their influence on CHD risk [4], [5], [17]. Finnish men who had a high ponderal index at birth had a relative risk of CHD of less than 0.75 even if they became relatively overweight in childhood, whereas the increased CHD risk associated with an increase in BMI in childhood was confined to men who were thin at birth (HR = 1.27 [95% CI: 1.10 to 1.47] per BMI standard deviation score) [4], [5]. The highest death rates from CHD occurred in men who were thin at birth, but whose weight caught up so that they had an average or above average BMI at age 11 years (HR = 5.3 [95% CI: 1.7 to 16.1]) [4], [5]. Recently, another Danish study has shown that birth weight and BMI in young adulthood seem to interact on the risk of CHD; the CHD risk was inversely related to birth weight among men who were overweight in young adulthood (p = 0.04), and highest in any group when combined with low birth weight (HR = 5.19 [95% CI: 2.13 to 12.62]) [17]. Among men of normal weight, there was also an inverse relation at low birth weight, but in addition an increased risk at higher birth weights (HR = 1.39 [95% CI: 1.07 to 1.78] for birth weight >4.0 kg) [17]. A U-shaped relationship between birth weight and risk of coronary artery disease after adjustment for adult BMI has likewise been observed in Icelandic women, but not in men, among whom the relation was inverse [7]. However, most studies have reported an inverse association between birth weight and risk of CHD [2]–[6], [8], [12], [20]–[22].

Information on birth weight in the Finnish cohort and on BMI and CHD in both cohorts was for the present study collected prospectively. Birth weight was in the Danish cohort reported by the parents. This method has been shown to be valid [38], [39]; any imprecision in the recall would likely introduce random error and attenuate the results. The observed association between birth weight and CHD may be an epiphenomenon induced by confounding factors, which are related both to birth weight and CHD risk. Adjustment for year of birth essentially did not alter the CHD risk and distribution of birth weight changed very little in Denmark during the study period [18], which is why these factors are unlikely to account for the observed association. Other studies on birth weight and CHD found that the association was strengthened by adjustment for gestational age indicating that the intrauterine growth pattern has a stronger association to CHD than the birth weight per se [4], [40]. Thus, had an examination of gestational age been possible, a stronger association might have been identified.

One study showed that the effect of weight at one year on the risk of CHD was not accounted for by parental social class [10]. Furthermore, in studies where information on adult socio-economic status or adult lifestyle factors were available, adjustment had little effect on the independent association between birth weight and CHD [11], [17], [22], [41].

The inverse association between fetal growth and risk of CHD may have several explanations, but two phenomena have received particular attention in the continued investigation of the DOHaD hypothesis [42]. The first is developmental plasticity, which leads to permanent changes in the body's structure and function due to the intrauterine and early postnatal environment [2], [43]. The second is ‘compensatory growth’, occurring when undernutrition during early development is followed by rapid childhood growth [5]. Reduced fetal growth is associated with later insulin resistance and type 2 diabetes [44], both of which may mediate the link between low birth weight and CHD [41]. Babies born small who gain much weight may have a disproportionately high fat mass [45]. Studies suggest that childhood growth influences cardiovascular risk factors, in particular impaired glucose regulation and lipid metabolism [46], but the importance of these findings for later health risks is uncertain [41]. In a Finnish cohort, rapid gain in BMI from age two through 11 years was associated with insulin resistance in later life [11]. Over an eight year follow-up, adults born small for gestational age gained more BMI than those born appropriate for gestational age, despite their lower likelihood of becoming obese, resulting in greater fat mass with more abdominal fat [47]. Small body size at birth and excessive weight gain during adolescence and young adulthood may predispose to low-grade inflammation in adulthood [48], which in turn also may increase the risk of developing CHD.

Although the change in the association from the inverse relation below 4 kg to apparently no association or, as seen in other studies, even an increase in CHD risk at higher birth weights could not be attributed to a relation to higher childhood BMI, there could still be a mixture of opposite effects. Thus, a possible tendency to further reduction in the risk of CHD by increasing birth weight may have been counterbalanced by high birth weights occurring as a consequence of maternal diabetes or its preceding stages, possibly developed during gestation. This may be associated with later increased offspring risk of diabetes [44], and metabolic syndrome [49]. Also, it may have been counterbalanced by a lower probability among subjects with high birth weight to undertake leisure time physical activity in adulthood [50], which is an important modifiable behavioural risk factor for CHD [51].

In conclusion, independently of BMI at age seven years, birth weights below 3.4 kg are inversely related to the risk of CHD in adult life, whereas at birth weights above this level the risk weakens, and above 4 kg birth weight is not associated with the risk. Preventing CHD-events by modifying birth weight in the lower range through intervention is not yet possible and may not make sense, because birth weight may be only an indicator of some factors more directly linked to CHD risk. These factors should be searched for and may be modifiable. From a public health perspective we suggest that treatment and prevention of childhood obesity should be given high priority, especially in individuals with a birth weight below the average.
